# Differentially Expressed Gene Patterns in Ascarid-Infected Chickens of Higher- or Lower-Performing Genotypes

**DOI:** 10.3390/ani11041002

**Published:** 2021-04-02

**Authors:** Isabel Kilic, Manuel Stehr, Mark Hennies, Cornelia C. Metges, Sören Franzenburg, Clemens Falker-Gieske, Jens Tetens, Gürbüz Daş

**Affiliations:** 1 Department of Animal Sciences, University of Göttingen, 37077 Göttingen, Germany; isabel.kilic@uni-goettingen.de (I.K.); clemens.falker-gieske@uni-goettingen.de (C.F.-G.); jens.tetens@uni-goettingen.de (J.T.); 2 Institute of Nutritional Physiology ‘Oskar Kellner’, Leibniz Institute for Farm Animal Biology, 18196 Dummerstorf, Germany; M.Stehr@ceravis.de (M.S.); metges@fbn-dummerstorf.de (C.C.M.); 3 TECOdevelopment GmbH, Marie-Curie-Str. 1, 53359 Rheinbach, Germany; hennies@tecodevelopment.com; 4 Institute of Clinical Molecular Biology, Christian-Albrechts-University of Kiel, 24105 Kiel, Germany; s.franzenburg@ikmb.uni-kiel.de; 5 Center for Integrated Breeding Research, University of Göttingen, 37075 Göttingen, Germany

**Keywords:** host-parasite interaction, host performance, nematode infection, peripheral blood, tolerance, transcriptome

## Abstract

**Simple Summary:**

Nematode infections may increase mortality and welfare problems in laying hens. The two ascarid worms, *Ascaridia galli* and *Heterakis gallinarum*, are highly prevalent in laying hens kept in non-cage housing systems worldwide. The ability of a host to expel pathogens is a component of resistance to diseases. The molecular basis of differences between different host animals in their efficiency to expel worms is, however, not well understood. Therefore, we performed a detailed analysis of differentially expressed genes (DEGs) in two chicken genotypes (Lohmann Brown Plus (LB), Lohmann Dual (LD)), each with a lower or higher infection intensity level of *A. galli* and *H. gallinarum.* Our data showed significant upregulation of *Guanylate Binding Protein 7 (GBP7)* in LD hens. Gene ontology analysis revealed higher transcriptome activity related to “response to external stimulus” in LB hens, implying a higher stress response in this genotype. In contrast, LD hens showed higher transcriptomic expression of genes associated with a higher tolerance to infections.

**Abstract:**

Here, we describe the first transcriptomic investigation of the peripheral blood of chickens exposed to *Ascaridia galli* and *Heterakis gallinarum* infections. We investigated differentially expressed gene (DEG) patterns in two chicken genotypes with either a higher (Lohmann Brown Plus, LB) or lower (Lohmann Dual, LD) laying performance level. The hens were experimentally coinfected with *A. galli* and *H. gallinarum*, and their worm burdens and infection parameters were determined six weeks post infection. Based on most representative infection parameters, the hens were clustered into lower- and higher-infection intensity classes. We identified a total of 78 DEGs contributing to infection-related phenotypic variation in the two genotypes. Our data showed significant upregulation of *Guanylate Binding Protein 7* (*GBP7*) in LD hens, making it a promising candidate for tolerance to ascarid infections in chickens. Gene ontology analysis revealed higher transcriptome activity related to biological processes such as “response to external stimulus” in LB hens, implying a higher stress response in this genotype. In contrast, LD hens showed higher transcriptomic expression of genes related to ontology classes that are possibly associated with a higher tolerance to infections. These findings may help explain why lower-performing genotypes (i.e., LD) are less sensitive to infections in terms of maintaining their performance.

## 1. Introduction

Nematode infections with the highly prevalent species *A. galli* and *H. gallinarum* [1,2,3,4] are associated with increased mortality and welfare problems in laying hens kept in non-cage housing systems [5]. The infections impair host animal performance and affect economically important egg-quality traits, mainly through reduced feed intake and/or deteriorating feed conversion efficiency [6,7,8,9].

The ability of a host to expel pathogens is a component of resistance to diseases [10]. Both resistance and tolerance to parasite infections are considered important functional traits in farm animals [11]. Several studies have shown host genotype effects on response to infections and metabolic disorders in chickens (e.g., [12,13]). Host genotype-dependent differences in resistance to nematode infections in chickens have also been described [14,15,16]. The molecular basis of differences due to the genetic background of host animals, which may elucidate how some individuals/genotypes are able to expel their worms more efficiently than others is, however, not well understood. Tolerance to nematode infections is also linked with the host genotype in chickens, but is also associated with host animal performance levels [8,9]. Following nematode infections, laying performance was impaired immediately in a higher-performing genotype (Lohmann Brown Plus, LB), whereas a lower-performing genotype (Lohmann Dual, LD) was able to tolerate adverse effects of infection on performance during the first weeks of infection [8]. The relationship between performance-level and tolerance to nematode infections was also shown to exist for growing male birds of divergently selected lines [9]. Thus, collective data on performance-level-dependent tolerance to nematode infections suggest that high-performing hosts are less tolerant to nematode infections. Recently, it was demonstrated that experimentally infected chickens are able to expel the vast majority of ascarids (i.e., *A. galli* and *H. gallinarum*) within a few weeks, particularly when the worms are in juvenile stages [17]. Nevertheless, considerable variation exists among individual chickens in their capability to expel worms, as the final worm burdens of experimentally infected birds vary greatly. The variation in both tolerance to infection and worm burdens of chickens may indicate the existence of associated variability in key factors involved in pathways responsible for immune functions and physiological processes to resolve nematode infections.

Gene expression patterns in cells of the infected host animal may provide information crucial to gaining a comprehensive understanding of the molecular regulation of immune response and physiological processes. In this respect, transcriptome data are particularly insightful, as they provide direct information related to the functional part of the host genome [18]. Avian nucleated red blood cells (RBCs) contain transcriptional and translational requirements for producing the characteristic molecules of the immune system to defend against pathogenic attacks. Therefore, nucleated RBCs seem to be involved in the regulation of both innate and adaptive immune responses, which highlights the crucial role of RBCs in host defence against pathogens [19]. To the best of our knowledge, there are no reports on gene expression in peripheral blood cells in nematode-infected chickens with different genetic backgrounds. Therefore, the aim of this study was to investigate transcriptomic differences in RBCs of ascarid-infected chickens of two genotypes, each with a lower or higher infection intensity level. For this purpose, we performed a detailed analysis of differentially expressed gene (DEG) patterns of the two chicken genotypes with either lower or higher infection phenotypes, using a large panel of variables serving as direct or indirect infection proxies.

## 2. Materials and Methods

### 2.1. Ethics Statement

Ethical approval for the experiment was obtained from the relevant state ethics committee for animal experimentation (Mecklenburg-Western Pomerania State Office for Agriculture, Food Safety, and Fisheries, Germany; permission no.: AZ.: 7221.3-1-080/16). The experiment was conducted in accordance with animal welfare rules (animal care and handling, stunning, and necropsies), and all sampling procedures were performed by trained/authorised staff. Experimental infection procedures were in line with the relevant guidelines of the World Association for the Advancement of Veterinary Parasitology for Poultry [20].

### 2.2. Animals, Management, Experimental Infection, and Sample Collection

For the present study, we used 12 hens of two genotypes, namely, Lohmann Brown Plus (LB, *n* = 7) and Lohmann Dual (LD, *n* = 5). The two genotypes differ considerably in their performance levels. While LB is a typical high-performing layer genotype, LD has been developed to produce dual-purpose animals, with females used for laying and males for fattening purposes so that culling of male birds may be redundant [8,9]. In the pre-experimental period (24 weeks), the hens were kept in helminth-free conditions. The hens were vaccinated against major bacterial and viral diseases (e.g., Salmonella, Newcastle disease, and infectious bronchitis) as well as coccidiosis (Paracox 8) at the recommended ages at the Farm for Education and Research Ruthe, University of Veterinary Medicine Hannover, Foundation, Germany. During the experimental period, the hens received no further vaccinations or medical treatment. The hens were fed a commercial layer diet that contained 11.2 MJ of metabolisable energy, 170 g of crude protein, and 3.6 g of calcium per kg feed (i.e., as-fed basis). Feed and water were offered for ad libitum intake. The lighting (light: 14 h; light intensity: 10–15 lux) and temperature (18–20 °C) regimes were as suggested by the breeding company. The hens were kept in a room with a floor husbandry system, using wood shavings as litter material.

When the hens were at the beginning of the laying period (i.e., 24 weeks old), experimental (co)infection of chickens with *A. galli* and *H. gallinarum* was performed. Origin of the nematode eggs, incubation conditions for embryonation, and preparation of the final infection inoculum have been described in detail elsewhere [17]. Incubated nematode eggs were assessed to determine the percentage of fully embryonated eggs that were considered infectious [21]. On the day of infection, separately incubated eggs of *A. galli* and *H. gallinarum* were merged in a final dose of 0.4 mL/hen containing 1000 embryonated eggs of the two species in equal proportions (i.e., 500 eggs per worm species). Hens were given the infection dose orally by using a 5-cm oesophageal cannula. 

### 2.3. Phenotyping of Infection Intensity through Direct Measurements of Worm Burdens

At 6 weeks post infection (wpi), the hens were necropsied to quantify their worm burdens with either nematode species. Immediately post mortem, the gastrointestinal tract was removed, and the small intestine and caeca were isolated to quantify worm burdens with *A. galli* and *H. gallinarum* in their predilection sites, respectively. For *A. galli*, the small intestine was opened longitudinally, and the intestinal content was washed through a sieve (36 µm) under running water. Tissue-associated *A. galli* larvae were recovered by using a slightly modified EDTA incubation method [17,22,23]. In short, after removing the luminal contents, the intestinal tissue was squeezed through a pair of pencil pincers under running lukewarm tap water to remove accidentally attached luminal worms. Immediately following this step, the washed tissue was hung into a preheated 400 mL EDTA solution (10 mM EDTA, 0.9% NaCl) for overnight incubation (>22 h at 40 °C). Thereafter, the EDTA solution was passed through a 20 µm sieve to collect the tissue larvae.

*H. gallinarum* was harvested from the caecal lumen contents by applying the same procedure as described for *A. galli* above. The caecal tissue and lumen contents were flushed under running water through a sieve (20 µm) to isolate immature and mature *H. gallinarum*. Worms of both species collected from each chicken were then placed in Petri dishes for counting, sex differentiation, and length measurements using a stereomicroscope at 40× magnification.

### 2.4. Phenotyping of Infection Parameters and Non-Specific Immunoglobulins

One day prior to necropsies, the hens were kept in single cages to collect individual chicken eggs and faecal droppings over 24 h. Faecal egg counts (FECs) describing both the nematode egg concentration in faeces (eggs per gram of faeces, EPG) and the total egg excretion within 24 h (eggs per day, EPD) were quantified. The daily total faeces was thoroughly mixed, and a random sub-sample (2 g) was analysed with the Mini-FLOTAC egg counting technique [24] using a saturated sodium chloride solution as the flotation liquid (specific gravity = 1.2). The minimum detection level of the Mini-FLOTAC technique was 10 eggs/g faeces. After quantification of the nematode egg concentration in faeces (EPG), the total number of eggs excreted within 24 h from each hen was estimated by multiplying the amount of total daily faeces with the EPG. Eggs of *A. galli* and *H. gallinarum* were not differentiated and counted together since they cannot be reliably differentiated [25]. An in-house-tailored enzyme-linked immunosorbent assay (ELISA) was used to quantify antigens of *A. galli* in the host faeces.

Slaughter blood was collected from the hens in both potassium-EDTA (Kabe Labortechnik GmbH, Nümbrecht-Elsenroth, Germany) and RNAlater tubes (400 µL of blood + 1.3 mL of RNAlater; Sigma Aldrich Chemie GmbH, Nümbrecht-Elsenroth, Germany). For quantification of ascarid-specific IgY (Asc-IgY) in plasma, blood was centrifuged at 2500 g for 20 min, and the supernatant was stored at −20 °C for later analysis. The chicken eggs were opened, and egg yolks were collected. A sub-sample of the egg yolks (250 µL) was diluted with 1.5 mL of purified water (pH = 2.5) and homogenised by using a vortexer. Egg yolk samples were then centrifuged at 12,000 g for 15 min. Asc-IgY levels in plasma (Asc-IgY-P) and egg yolk (Asc-IgY-EY) samples were then determined with an ELISA as previously described [26]. The laboratory-specific intra- and inter-assay coefficients of variability (CVs) for the assay were 5.0% and 8.4%, respectively.

Concentrations of immunoglobulins IgY (IgY-P), and IgM in plasma (IgM-P) samples were determined using commercial ELISA kits (IgY: Kit No. E30-104; IgM: Kit No. E30-103; Bethyl Laboratories, Inc., Montgomery, TX, USA). The laboratory-specific intra-assay CV and inter-assay CV for the analysis ranged between 5.0 and 7.6% and 7.7 and 10.4%, respectively.

### 2.5. Infection Intensity Clusters

A principal component analysis (PCA) followed by a cluster analysis (CA) of variables that directly or indirectly reflected the infection intensity of chickens with both nematodes was performed to classify hens into lower- or higher-infection intensity classes. For this purpose, in addition to the direct worm burdens with *H. gallinarum* and *A. galli*, the total number of nematode eggs excreted within 24 h through the host faeces (i.e., EPD) and *A. galli* antigen concentration in faeces were also used as independent variables for both analyses. The PCA and CA were performed using JMP15 (https://www.jmp.com/support/help/en/15.2/; access date: 12 December 2020). The analyses resulted in two distinct classes of chickens differing in overall infection intensity levels, i.e., lower and higher (Supplementary Figure S1). In the first cluster (lower infection intensity), there were seven hens, four of which were LB hens and three of which were LD hens. In the second cluster (higher infection intensity), there were five hens (3 LB and 2 LD). The proportion of variation explained by the two clusters was approximately 76.2%.

### 2.6. Blood RNA Isolation and Quality Assessment

Total RNA was extracted using TRIzol Reagent (Life Technologies, Carlsbad, CA, USA). The protocol was followed according to the manufacturer’s recommendation, with minor modifications: each sample (total volume (blood + RNAlater) ~1.5 mL) was divided into three aliquots and centrifuged for 1 min at 21,000 g and 4 °C to remove the RNAlater. Phase separation was achieved by 30 min of centrifugation at 12,000 g and 4 °C.

Total blood RNA was precipitated in ice-cold isopropyl alcohol and then washed in 300 µL of 80% ethanol. RNA pellets were eluted in 11 µL of RNase-free water (Thermo Fisher Scientific, Waltham, MA, USA). The RNA concentration was measured at a wavelength of 260 nm, and the purity of RNA was assessed by the absorbance at 230 and 280 nm with a NanoPhotometer^®^ P-360 spectrophotometer (Implen, Munich, Germany). RNA pellets received from one blood sample were then pooled and DNase I digested using an RNA Clean & Concentrator-5 Kit (Zymo Research Corp., Irvine, CA, USA) according to the manufacturers recommendations.

RNA quantity and quality were measured using a Qubit 2.0 fluorometer (Thermo Fisher Scientific, Waltham, MA, USA) and microchip electrophoresis on a TapeStation (Agilent, Santa Clara, CA, USA). The RNA integrity number (RIN) of most samples was >7.

### 2.7. Library Preparation and RNA Sequencing

Sample library preparation was performed from 100–500 ng of total RNA input using the TruSeq Stranded mRNA Kit following the manufacturers’ recommendations. The library preparations were sequenced on an Illumina NovaSeq 6000 SP platform (Illumina Inc., San Diego, CA, USA), aiming for 25 million 2 × 50 bp paired-end reads per sample. This was followed by FASTQ file generation.

### 2.8. Transcriptome Analyses

Raw sequencing reads were processed with Trimmomatic version 0.36 [27] for adapter removal, trimming of low-quality base calls, and removal of low-quality reads. Trimmomatic was used with the following settings: PE -phred33 LEADING:3 TRAILING:3 SLIDINGWINDOW:4:15 MINLEN:36. Read pairs were discarded if one read did not survive quality control. Trimmed reads were aligned to chicken genome version GCF_000002315.5 (RefSeq assembly, downloaded from Ensembl (http://www.ensembl.org/index.html, accessed on 6 June 2018) using TopHat version 2.1.0 [28] with the settings --no-novel-juncs --min-isoform-fraction 0.0 --min-anchor-length 3 -r 192 and GCF_000002315.5.gff as the known transcript file. Genomic features were extracted from the general feature format file and grouped with the R package GenomicFeatures version 1.36.4 [29]. The summarizeOverlaps function in the R package GenomicAlignments version 1.20.1 was used to count exon-spanning reads [29].

Differential gene expression was analysed with DESeq2 (version 1.22.2) [30]. We conducted four separate comparisons to estimate the effects of (I) host genetic background (LD vs. LB); (II) nematode infection intensity level (lower vs. higher); (III) host genetic background adjusted for nematode infection intensity level; and (IV) nematode infection intensity level adjusted for genotype effects.

The fold changes (on a log2 scale) and *p*-values of the differentially expressed genes (DEGs) were acquired in the output files from DESeq2. Adjusted *p*-values were obtained using the Benjamini and Hochberg method, and an adjusted *p*-value of 0.05 and a log2 fold change of 1.0 were assigned as thresholds for significant differential expression. Volcano plots of differential expression analyses were created with the R package EnhancedVolcano (version 1.2.0) [31].

### 2.9. Gene Set Enrichment (GSE) and Pathway Analysis of DEGs

DEGs were functionally annotated and further analysed via gene set enrichment and pathway analysis using the R package gprofiler2 (version 0.1.7) [32]. KEGG pathways (https://www.genome.jp/kegg/pathway.html accessed on 19 November 2020) with *p* < 0.05 were considered significantly enriched among the differentially expressed genes. The analyses were performed separately for each of the four abovementioned comparisons.

### 2.10. Statistical Analysis of the Phenotypic Data

The two genotypes were compared for direct (i.e., number of worms per hen) and indirect infection parameters (e.g., nematode egg excretion and antibody levels in host plasma and egg yolks) using one-way ANOVA through the GLM (General Linear Models) procedure in SAS/STAT (version 9.4) software of the SAS System for Windows (SAS Institute Inc., Cary, NC, USA). With the exception of Asc-IgY-P (*p* = 0.041), all other variables showed normal distributions when tested with the Kolmogorov-Smirnov test (*p* > 0.05). Since the Shapiro—Wilk test indicated a normal distribution for Asc-IgY-P (*p* = 0.095), all data were considered as normally distributed.

Pearson’s correlation coefficients were calculated to examine the relationships between different infection parameters using pooled data (*n* = 12). Correlation analysis and data visualisation were performed using the R packages Hmisc (version 4.3-1) [33] and ggplot2 (version 3.2.1) [34].

## 3. Results

### 3.1. Phenotypic Traits

As shown in Table 1, there was no significant difference (*p* > 0.05) between the two genotypes in their total worm burdens with *A. galli* or *H. gallinarum* at 6 wpi. Nematode egg excretion through host animal faeces, quantified as egg concentration (EPG) or total egg number per day (EPD), did not differ between the two host genotypes (*p* > 0.05). Similarly, the levels of ascarid-specific IgY in plasma or in egg yolks did not differ significantly between the two genotypes (*p* > 0.05). Ascarid antigens quantified in the faeces of hens were not influenced by host genotype (*p* > 0.05). Concentrations of non-specific IgY and IgM in plasma did not differ between the two genotypes (*p* > 0.05), while LB hens tended to have a higher non-specific IgY concentration in egg yolks than did LD hens (*p* > 0.05).

**Figure 1:**
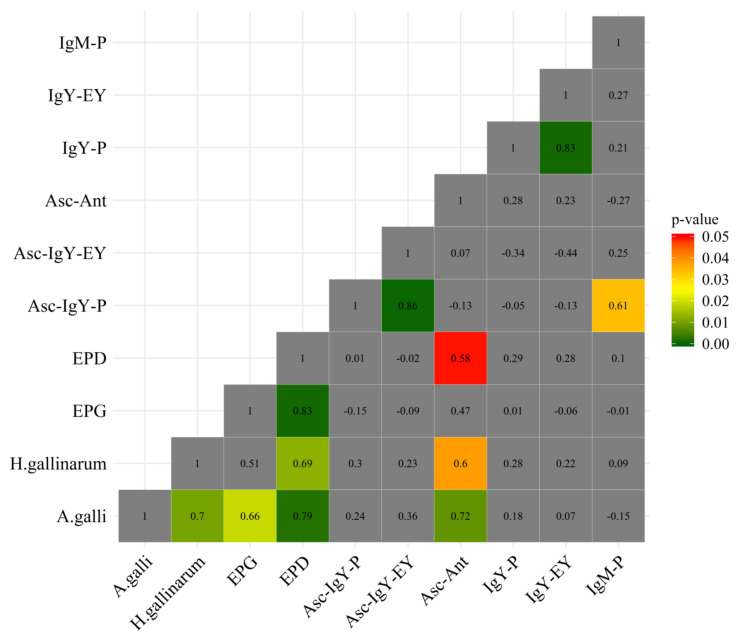
Heatmap of associations between phenotypic traits included in the study. Values shown in the boxes are Pearson’s correlation coefficients. Non-grey-coloured boxes indicate significant correlations.

The estimation of associations between different phenotypic traits revealed significant correlations between the numbers of *A. galli* and *H. gallinarum* worms (r = 0.70; *p* = 0.01; Figure 1). The number of *A. galli* correlated positively with EPG (r = 0.66; *p* < 0.05), EPD (r = 0.79; *p* < 0.05) and Asc-Antigen (r = 0.72; *p* < 0.01). Furthermore, the number of *H. gallinarum* positively correlated with EPD (r = 0.69; *p* = 0.01) and with Asc-Antigen (r = 0.60; *p* < 0.05). Concentrations of ascarid-specific IgY in plasma and egg yolks were correlated strongly and positively (r = 0.86, *p* < 0.001). Further correlations are presented in Figure 1.

### 3.2. RNA Sequencing and Differential Gene Expression Analysis

RNA-Seq technology was used to investigate differences in gene expression that could be involved in the molecular regulation of worm expulsion and tolerance to nematode infection. After removing adaptors and low-quality reads, we obtained a total of 968,891,196 high-quality reads, with an average of 80,740,933 (range: 29,965,120 to 148,212,019 reads) for each sample. Alignment of the sequence reads against the chicken reference genome (GRCg6a) using TopHat yielded 83.1 to 95.4% aligned reads across all samples (Supplementary Table S1).

A total of 24,021 genes were read in RNA-Seq expression profiles, with 20,250 expressed transcripts in the peripheral blood of the sampled hens (Supplementary Table S2). Differential gene expression analysis revealed a total of 78 significantly differentially expressed genes (log2FC ≥ 1.0, padj < 0.05) across the four abovementioned comparisons (I–IV).

**Figure 2:**
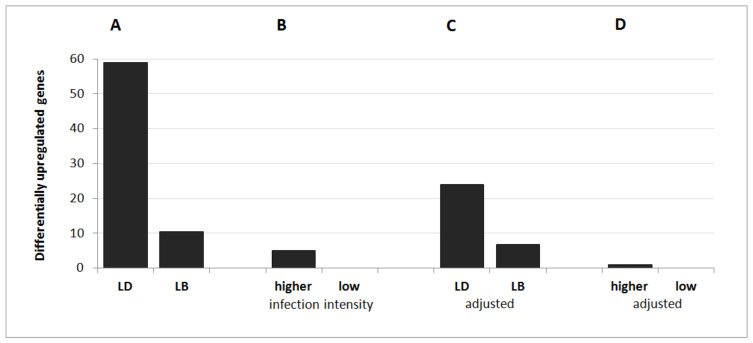
Number of significantly upregulated genes (**A**) between Lohmann Dual (LD) and Lohmann Brown Plus (LB) hens, (**B**) between hens with lower and higher infection intensity levels, (**C**) between LD and LB genotypes adjusted for infection intensity, and (**D**) between lower and higher infection intensity levels adjusted for genotype effects.

**Figure 3:**
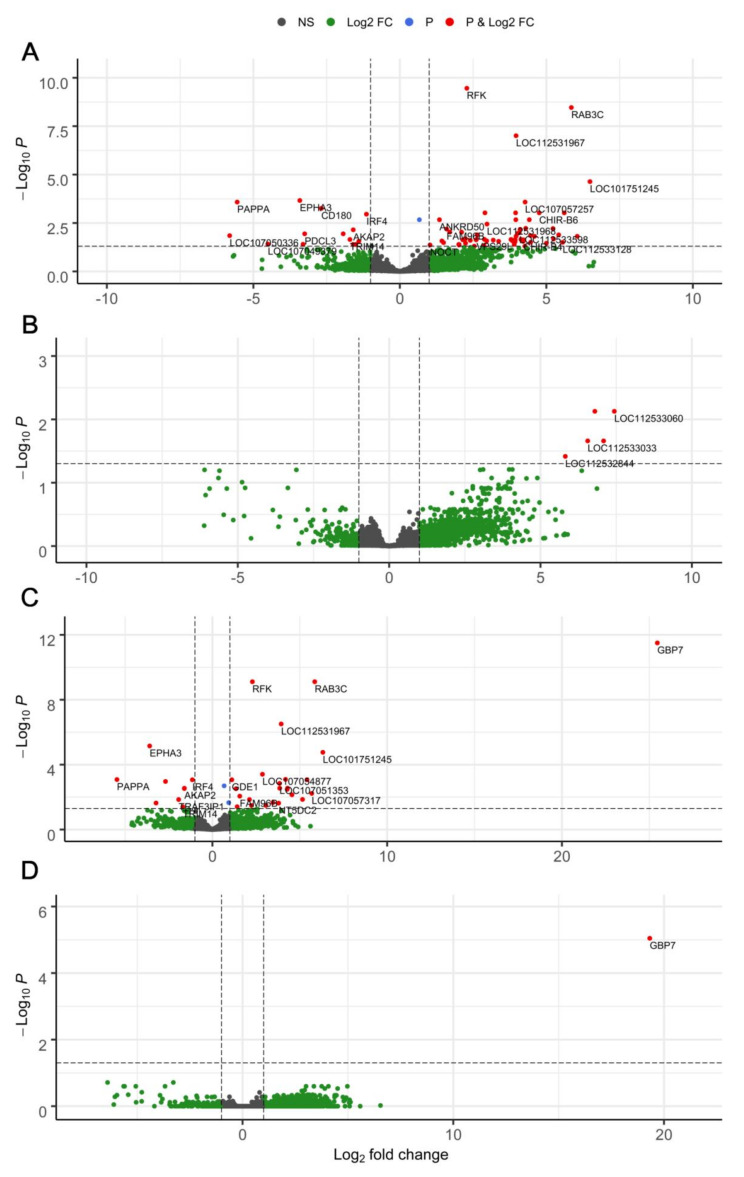
Volcano plots illustrating significantly differentially expressed genes (red dots) influenced by (**A**) host genetic background (Lohmann Dual vs. Lohmann Brown Plus); (**B**) nematode infection intensity level (lower vs. higher); (**C**) the effect of host genetic background adjusted for infection intensity level; and (**D**) the effect of infection intensity adjusted for genotype effects. Abbreviations: NS: not significantly different; Log2FC: significantly different according to Log2FC; P: significantly different according to *p*-value; P and Log2FC: significantly different according to p-value and Log2FC.

In the first comparison (I), 59 genes were upregulated and 14 were downregulated in LD hens compared to LB hens (Figure 2A). Among those genes, 33 (30 upregulated and 3 downregulated) were of uncertain function (LOC symbols). Comparison II revealed five LOCs, which were significantly upregulated in hens with higher versus lower infection intensity (Figure 2B). Regarding the effect of host genetic background adjusted for infection intensity level (comparison III), a total of 33 genes, including 12 LOCs, were differentially expressed (Figure 3C). Twenty-four of these DEGs were significantly upregulated and nine were downregulated in LD hens compared to LB hens (Figure 2C and Figure 3C). We identified one DEG between hens with a lower infection intensity level compared to those with a higher infection intensity level. This DEG was upregulated in hens with higher infection intensity levels (Figure 2D).

Figure 3 illustrates the results for significantly DEGs out of 24,021 genes with respect to host genetic background (Figure 3A), nematode infection intensity level (Figure 3B), the effect of host genetic background adjusted for infection intensity level (Figure 3C) and the effect of infection intensity adjusted for host genotype effects (Figure 3D). Regarding the differential gene expression between LD and LB hens (I), *Riboflavin kinase* (*RFK*, log2FC = +2.28, *p* < 0.001) and *Ras-related protein Rab-3C* (*RAB3C*, log2FC = +5.85, *p* < 0.001) were the most significantly DEGs in LD hens (Figure 3A). In contrast, *Ephrin type-A receptor 3* (*EPHA3*, log2FC = −3.59, *p* < 0.001), *Pappalysin-1* (*PAPPA*, log2FC = −5.46, *p* < 0.001), and *CD180 molecule* (*CD180*, log2FC = −2.68, *p* < 0.005) were significantly downregulated in LD hens compared to LB hens (Figure 3A). There were no annotated DEGs directly affected by infection intensity level, whereas five upregulated LOCs (e.g., *LOC112533060*, log2FC = +7.44, *p* < 0.01; *LOC112533033*, log2FC = +6.56, *p* < 0.05) were identified in hens with higher infection intensity (Figure 3B). Regarding the effect of host genetic background adjusted for infection intensity level (Figure 3C, i.e., comparison III), most of the identified DEGs were similar to those affected by host genotype only (Figure 3A), although the top DEG between LD and LB hens was *Guanylate binding protein 7* (*GBP7*, log2FC = +25.46, *p* < 0.001). Analysing DEGs based on the effect of infection intensity adjusted for genotype effects (IV), we found significant upregulation of *GBP7* (log2FC = +19.32, *p* < 0.001) in hens with higher infection intensity (Figure 3D).

### 3.3. Gene Ontology Enrichment Analysis

Gene ontology (GO) term enrichment analysis was performed to obtain a better understanding of the functions of differentially expressed genes. Of all identified DEGs, a total of 52 genes (39 upregulated and 13 downregulated) were annotated. Annotations of upregulated DEGs in LD and LB hens are listed in Table 2 and Table 3, respectively.

**Figure 4:**
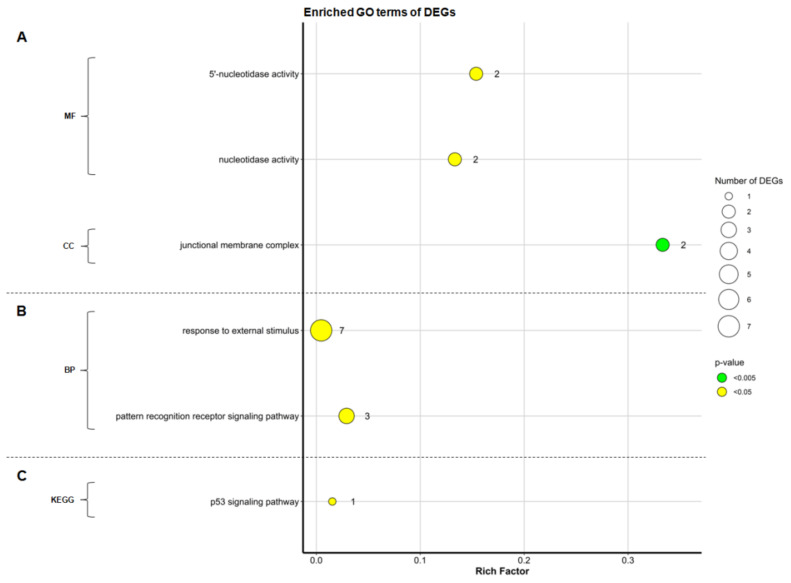
Gene ontology (GO) enrichment and KEGG pathway analysis based on differentially expressed genes (DEGs between ascarid-infected Lohmann Dual (LD) and Lohmann Brown Plus (LB) hens. Panel (**A**) shows enriched GO terms derived from upregulated DEGs in LD hens without (I) or with (III) regard to infection intensity*; Panel (**B**) represents upregulated DEGs in LB hens (I); Panel (**C**) represents the result of KEGG pathway analysis derived from upregulated DEGs in LB hens without (I) and with (III) regard to infection intensity *. The vertical axis represents the enriched GO terms, and the horizontal axis represents the affiliated rich factor (ratio of the number of DEGs associated with the GO term to the total number of genes associated with the GO/KEGG term). The size and colour of dots represent the number of DEGs and the range of adjusted *p*-values (after Bonferroni correction), respectively. MF: molecular function; CC: cellular component; BP: biological process; *: DEGs identified in comparisons I and III resulted in the same GO patterns.

The DEGs were classified under three major categories of GO terms: “molecular function” (MF), “cellular component” (CC), and “biological process” (BP). Figure 4 shows the results of GO term and KEGG pathway enrichment analysis for the comparisons of LD and LB hens without (I) or with (III) adjustment for infection intensity.

Upregulated genes in LD hens (comparisons I and III) were assigned to the molecular functions “5′-nucleotidase activity” (GO:0008253) and “nucleotidase activity” (GO:0008252). Another GO term, “junctional membrane complex” (GO:0030314), was significantly enriched in the CC category (Figure 4A), including *Junctophilin 2* (*JPH2*).

Among the annotated upregulated DEGs in LB hens (comparison I), two GO terms were significantly (*p* < 0.05) enriched in the BP category (Figure 4B). The DE genes *EPHA3*, *CD180*, *IRF4*, *TRAF3IP1*, *TRIM14*, *DDX60,* and *ANO6* were enriched in “response to external stimulus” (GO:0009605). *CD180*, *IRF4,* and *DDX60* were assigned to “pattern recognition receptor signaling pathway” (GO:0002221).

Furthermore, in LB hens (comparison I), genes linked to “p53 signaling pathway” (KEGG:04115) were significantly enriched in KEGG pathway analysis (Figure 4C), which was also the case when taking infection level into account (comparison III).

There was no GO enrichment of DEGs derived either from the comparison of lower and higher infection intensities (II) or from the comparison of infection intensities adjusted for genetic background (IV).

## 4. Discussion

The aim of this study was to identify genes and molecular pathways that are potentially involved in the defence function and physiological processes in nematode-infected chickens of two genotypes with different performance levels. We used cohorts of infected LB and LD hens, whose pen-based performance data and worm burdens over a period of 18 weeks were reported in detail by Stehr et al. [8]. Up to the time of sampling in the present study (i.e., 6 wpi), there was no difference between the two genotypes in their worm burdens resulting from experimental infections, which was also confirmed with the data for the present cohorts (Table 1). In the same period of time (6 weeks), the laying performance of LB hens was adversely affected by the infections, whereas LD animals showed no impairment in their laying performance, suggesting a higher tolerance to primary nematode infections [8]. Since the two genotypes were highly similar in terms of total worm burdens at 6 wpi, differences in the transcriptomic profiles of the chickens may instead be related to their ability to withstand the infections, i.e., tolerance to infections. To account for variability in worm burdens of hens within each genotype, we included infection intensity of the hens as an additional factor in the transcriptomic analysis. In the following sections, we discuss transcriptomic differences in the RBCs of infected hens of LB and LD genotypes with respect to tolerance and resistance to infections.

Several studies have shown that gene expression profiling of peripheral blood cells yields valuable diagnostic and prognostic information regarding various disease states [35,36,37,38]. Immune cells in the peripheral blood are migratory cells whose transcriptional profile may be influenced by the presence of pathogens [39]. Therefore, excess erythrocyte transcripts suggest their participation in the immune response [40]. We identified a total of 78 DEGs across four different comparisons (I-IV). The largest number of DEGs was associated with host genetic background (*n* = 74). After adjustment for infection intensity level, 33 DEGs were attributable to the genetic background of hens (LD vs. LB). These results indicate that differences in gene expression were largely due to the genetic background rather than the infection intensity of the host animal.

Based on the highly significant differential expression patterns, genes that might play a role in regulating performance-dependent tolerance to nematodes include *Guanylate Binding Protein 7* (*GBP7*), *Riboflavin kinase* (*RFK*), and *interferon regulatory factor 4* (*IRF4*). We identified *GBP7* as the major differentially expressed transcript between LD and LB hens when gene abundance was adjusted for infection intensity. Recently, guanylate-binding proteins (GBPs) were found to play essential roles in immunity to infection, inflammation, and neoplastic diseases [41,42]. Steffens et al. [41] demonstrated that *GBP7*^−/−^-mice show dramatic susceptibility and mortality after *Toxoplasma gondii* (*T. gondii*) infection. To the best of our knowledge, there are no studies examining the role of chicken GBPs during parasite infections. Our findings suggest the activation of *GBP7* after ascarid infections, which leads to the assumption that GBPs show strong functional similarities between birds and mammals. Our results are nevertheless limited to a period of 6 weeks post infection. Follow-up studies, including groups of uninfected control animals, may specifically focus on analysing *GBP7* and *IRF4* expression at several time points following infection.

Another crucial transcriptomic difference was observed in *RFK*, which was significantly upregulated in LD hens compared to LB hens. Activation of *RFK* indicates the effects of spatially and temporally controlled reactive oxygen species production, which plays important roles in, e.g., innate immunity or inflammatory diseases [43]. In contrast to *RFK*, *IRF4* was significantly upregulated in LB hens. The protein product of *IRF4* is required during the immune response for lymphocyte activation and the generation of immunoglobulin-secreting plasma cells [44]. Ruhnke et al. [45] showed a time-dependent increase in the number of intraepithelial CD4+ T helper cells in *A. galli*-infected broiler chickens, peaking at day 20 post infection. The time of onset of the adaptive immune response could be confirmed by elevated levels of ascarid IgY in our previous studies [17,46].

GO and KEGG analyses showed that the upregulated genes in LD hens were involved in catalytic processes or in intracellular anatomical structure, e.g., “junctional membrane complex”. These findings may support previous data [17] that the abundance of *Claudin-1* (*CLDN1*), a major constituent of tight junction complexes, is decreased in the jejunum of infected LB birds. The present study also indicates very low abundances of *CLDN1* in the samples of infected birds, although it was not differentially expressed in any comparisons (data not shown).

We detected enrichment of upregulated genes in LB hens that were involved in “response to external stimulus”. The higher gene abundances related to response to external stimulus in LB hens may imply a more pronounced stress response to ascarid infection in this genotype than in the other genotype. Furthermore, the higher response to external stimuli and the resulting stress might help explain the lower tolerance to infection in LB hens than in LD hens. Host responses to helminth infections are characterised by a trade-off between performance and immunity against the parasite caused by prolonged inflammatory responses. The host animal often favours tolerance over complete helminth destruction to limit immune-mediated damage [47]. Identifying genes and physiological mechanisms affecting tolerance is a key step in understanding the genetic and physiological bases of variation in tolerance. Tolerance genes and pathways are suggested to be involved in tissue repair and scavenging of damaging molecules produced during infection [48]. An important potential candidate gene is *GBP7*, which has already been described in connection with infections of parasites (*T. gondii*) [41], bacteria [49], or Influenza A virus [42] in mice and perhaps has a similar function in ascarid infections in chickens. 

## 5. Conclusions

This is the first transcriptomic investigation of the peripheral blood of chickens exposed to nematode infections, and it reveals specific genes related to performance-level-dependent tolerance to ascarid infections and response genes involved in these processes. We identified 78 DEGs contributing to infection-related phenotypic variation in two genotypes. The vast majority of the DEGs were associated with host genotype rather than infection intensity level. Our data showed significant upregulation of *GBP7* in LD hens, making it a promising candidate gene for tolerance to ascarid infections in chickens. Further, the most pronounced differences were observed for *RFK*, *IRF4,* and *EPHA3*, all of which are related to immune function and physiological processes in infected hosts. Gene ontology enrichment analysis revealed higher transcriptome activity related to biological processes such as response to external stimulus in LB hens than in LD hens, implying a more pronounced stress response in this genotype. In contrast, LD hens showed a higher abundance of genes related to molecular functions, cellular components, and biological processes, possibly associated with a higher tolerance to infections. These findings may help explain why lower-performing genotypes (i.e., LD) are less sensitive to infections in terms of maintaining their performance. Further studies, including groups of uninfected control animals, should particularly focus on the expressions of *GBP7*, *RFK*, *IRF4,* and *EPHA3* at several time points following infection. 

## Figures and Tables

**Table 1:** Direct and indirect infection parameters measured at 6 wpi in hens of LD and LB genotypes following experimental co-infection with *A. galli* and *H. gallinarum*.

	LD (*n* = 5) *	LB (*n* = 7) *	*p*-Value
*A. galli*, *n*/bird	15 ± 6.5	16 ± 5.5	0.901
*H. gallinarum*, *n*/bird	172 ± 41.6	164 ± 35.2	0.890
EPG	1266 ± 377	607 ± 318	0.211
EPD	111,248 ± 49,424	109,383 ± 41,771	0.978
Asc-IgY-P, mU/mL	29.10 ± 7.325	38.61 ± 6.190	0.345
Asc-IgY-EY, mU/mL	21.17 ± 7.351	24.06 ± 6.213	0.770
Asc-Antigen, ng/g	732 ± 248.1	775 ± 209.6	0.897
IgY-P, mg/mL	4.88 ± 0.851	5.91 ± 0.719	0.379
IgY-EY, mg/mL	5.32 ± 0.771	7.32 ± 0.652	0.076
IgM-P, mg/mL	0.58 ± 0.086	0.67 ± 0.073	0.424

* Values are least-square means ± standard errors. *p*-values were derived from one-way ANOVA with host genotype as the single factor. Worm burdens with *A. galli* and *H. gallinarum* include all worms of different developmental stages. Abbreviations: LD: Lohmann Dual; LB: Lohmann Brown Plus; wpi: weeks post infection; EPG: number of eggs per gram of faeces; EPD: number of eggs excreted within 24 h; Asc-IgY-P: ascarid-specific-IgY in plasma; Asc-IgY-EY: ascarid-specific-IgY in egg yolk; Asc-antigen: *A. galli*-specific antigens in faeces; IgY-P: non-specific IgY in plasma, IgY-EY: non-specific IgY in plasma; IgM-P: non-specific IgM in plasma.

**Table 2:** Annotated differentially upregulated genes in Lohmann Dual (LD) hens.

Initial Alias	Description
ANKRD50	ankyrin repeat domain 50 [Source: NCBI gene; Acc: 422663]
CHIR-B6	Gallus gallus immunoglobulin-like receptor CHIR-B6 (CHIR-B6), mRNA. [Source: RefSeq mRNA; Acc: NM_001318406; NCBI gene; Acc: 429646]
EFNB1	ephrin B1 [Source: NCBI gene; Acc: 395896]
EVA1C	eva-1 homolog C [Source: NCBI gene; Acc: 418496]
FAM96B	family with sequence similarity 96 member B [Source: NCBI gene; Acc: 415789]
GDE1	glycerophosphodiester phosphodiesterase 1 [Source: NCBI gene; Acc: 416612]
HCN3	hyperpolarization activated cyclic nucleotide gated potassium channel 3 [Source: NCBI gene; Acc: 100859704]
INTS7	integrator complex subunit 7 [Source: NCBI gene; Acc: 421374]
ITIH6	inter-alpha-trypsin inhibitor heavy chain family member 6 [Source: NCBI gene; Acc: 430871]
JPH2	junctophilin 2 [Source: NCBI gene; Acc: 770867]
LOC107049467	uncharacterized LOC107049467 [Source: NCBI gene; Acc: 107049467]
LOC107054877	uncharacterized LOC107054877 [Source: NCBI gene; Acc: 107054877]
LOC112532457	small nucleolar RNA SNORD17 [Source: NCBI gene; Acc: 112532457]
LOC112532735	Small nucleolar RNA SNORA74 [Source: RFAM; Acc: RF00090]
LOC112533044	5S ribosomal RNA [Source: RFAM; Acc: RF00001]
LOC112533070	5S ribosomal RNA [Source: RFAM; Acc: RF00001]
LOC112533128	5S ribosomal RNA [Source: RFAM; Acc: RF00001]
LOC112533129	5S ribosomal RNA [Source: RFAM; Acc: RF00001]
LOC112533523	U4 spliceosomal RNA [Source: RFAM; Acc: RF00015]
LOC112533598	5.8S ribosomal RNA [Source: RFAM; Acc: RF00002]
LOC112533600	5.8S ribosomal RNA [Source: RFAM; Acc: RF00002]
MAPK12	mitogen-activated protein kinase 12 [Source: NCBI gene; Acc: 769763]
MIR3528	gga-mir-3528 [Source: miRBase; Acc: MI0015379]
MRPS24	mitochondrial ribosomal protein S24 [Source: HGNC Symbol; Acc: HGNC:14510]
NOCT	nocturnin [Source: NCBI gene; Acc: 404779]
NT5DC2	5′-nucleotidase domain containing 2 [Source: NCBI gene; Acc: 415895]
NT5DC4	5′-nucleotidase domain containing 4 [Source: NCBI gene; Acc: 100858617]
OTX5	orthodenticle-related homeobox 5 [Source: NCBI gene; Acc: 103875463]
PABPN1L	poly(A) binding protein nuclear 1 like, cytoplasmic [Source: NCBI gene; Acc: 769028]
PLEKHH3	pleckstrin homology, MyTH4 and FERM domain containing H3 [Source: NCBI gene; Acc: 772127]
RAB3C	RAB3C, member RAS oncogene family [Source: NCBI gene; Acc: 770370]
RFK	riboflavin kinase [Source: NCBI gene; Acc: 427259]
SRXN1	sulfiredoxin 1 [Source: NCBI gene; Acc: 100858692]
SUOX	sulfite oxidase [Source: HGNC Symbol; Acc: HGNC:11460]
TCF15	transcription factor 15 (basic helix-loop-helix) [Source: NCBI gene; Acc: 395788]
TPRKB	TP53RK binding protein [Source: NCBI gene; Acc: 425906]
VPS29L	VPS29 retromer complex component-like [Source: NCBI gene; Acc: 416931]
WDFY2	WD repeat and FYVE domain containing 2 [Source: NCBI gene; Acc: 770107]
ZHX3	zinc fingers and homeoboxes 3 [Source: NCBI gene; Acc: 419176]

**Table 3:** Annotated differentially upregulated genes in LB hens.

Initial Alias	Description
AKAP2	PALM2-AKAP2 fusion [Source: NCBI gene; Acc: 100533110]
ANO6	anoctamin 6 [Source: NCBI gene; Acc: 417802]
CCNG1	cyclin G1 [Source: NCBI gene; Acc: 416161]
CD180	CD180 molecule [Source: NCBI gene; Acc: 431584]
DDX60	DExD/H-box helicase 60 [Source: NCBI gene; Acc: 422427]
EPHA3	EPH receptor A3 [Source: NCBI gene; Acc: 396402]
IRF4	interferon regulatory factor 4 [Source: NCBI gene; Acc: 374179]
LOC112533065	5S ribosomal RNA [Source: RFAM; Acc: RF00001]
LOC418108	poly [ADP-ribose] polymerase 12-like [Source: NCBI gene; Acc: 418108]
PAPPA	pappalysin 1 [Source: NCBI gene; Acc: 417245]
PDCL3	Phosducin-like 3 [Source: NCBI gene; Acc: 418709]
TRAF3IP1	TRAF3 interacting protein 1 [Source: NCBI gene; Acc: 424029]
TRIM14	tripartite motif containing 14 [Source: NCBI gene; Acc: 427282]

## Data Availability

Raw data are available from the authors upon request with a plausible reason.

## Supplementary Materials

The following are available online at https://www.mdpi.com/article/10.3390/ani11041002/s1; Figure S1: Principal component analysis (A-left panel) with loadings (A-right panel) and cluster analysis of variables that describe infection intensity of chickens with both nematodes (B), Table S1: Transcriptome alignment summary, Table S2: Results of differential gene expression analysis.

## References

[1] Kaufmann F., Daş G., Sohnrey B., Gauly M. (2011). Helminth infections in laying hens kept in organic free range systems in Germany. Livest. Sci..

[2] Wongrak K., Daş G., Moors E., Sohnrey B., Gauly M. (2014). Establishment of gastro-intestinal helminth infections in free-range chickens: A longitudinal on farm study. Berliner Münchener Tierarztliche Wochenschrift.

[3] Thapa S., Hinrichsen L.K., Brenninkmeyer C., Gunnarsson S., Heerkens J.L., Verwer C., Niebuhr K., Willett A., Grilli G., Thamsborg S.M. (2015). Prevalence and magnitude of helminth infections in organic laying hens (*Gallus gallus domesticus*) across Europe. Parasitology.

[4] Sharma N., Hunt P.W., Hine B.C., Ruhnke I. (2019). The impacts of Ascaridia galli on performance, health, and immune responses of laying hens: New insights into an old problem. Poult. Sci..

[5] Hinrichsen L.K., Labouriau R., Engberg R.M., Knierim U., Sørensen J.T. (2016). Helminth infection is associated with hen mortality in Danish organic egg production. Veter. Rec..

[6] Daş G., Abel H., Rautenschlein S., Humburg J., Schwarz A., Breves G., Gauly M. (2011). Effects of dietary non-starch polysaccharides on establishment and fecundity of *Heterakis gallinarum* in grower layers. Parasitology.

[7] Daş G., Abel H., Humburg J., Schwarz A., Rautenschlein S., Breves G., Gauly M. (2011). The effects of dietary non-starch polysaccharides on *Ascaridia galli* infection in grower layers. Parasitology.

[8] Stehr M., Grashorn M., Dannenberger D., Tuchscherer A., Gauly M., Metges C.C., Daş G. (2019). Resistance and tolerance to mixed nematode infections in relation to performance level in laying hens. Veter. Parasitol..

[9] Stehr M., Zentek J., Vahjen W., Zitnan R., Tuchscherer A., Gauly M., Metges C.C., Daş G. (2019). Resistance and tolerance to mixed nematode infections in chicken genotypes with extremely different growth rates. Int. J. Parasitol..

[10] Bishop S., Stear M. (2003). Modeling of host genetics and resistance to infectious diseases: Understanding and controlling nematode infections. Veter. Parasitol..

[11] Råberg L., Graham A.L., Read A.F. (2008). Decomposing health: Tolerance and resistance to parasites in animals. Philos. Trans. R. Soc. B Biol. Sci..

[12] Zhang J., Kaiser M.G., Deist M.S., Gallardo R.A., Bunn D.A., Kelly T.R., Dekkers J.C.M., Zhou H., Lamont S.J. (2018). Transcriptome analysis in spleen reveals differential regulation of response to Newcastle disease virus in two chicken lines. Sci. Rep..

[13] Blanco A., Barz M., Icken W., Cavero D., Mazaheri A., Voss M., Schmutz M., Preisinger R. (2016). Twenty years of amyloid arthropathy research in chickens. World Poult. Sci. J..

[14] Permin A., Ranvig H. (2001). Genetic resistance to *Ascaridia galli* infections in chickens. Veter. Parasitol..

[15] Kaufmann F., Daş G., Preisinger R., Schmutz M., König S., Gauly M. (2011). Genetic resistance to natural helminth infections in two chicken layer lines. Veter. Parasitol..

[16] Wongrak K., Daş G., Von Borstel U.K., Gauly M. (2015). Genetic variation for worm burdens in laying hens naturally infected with gastro-intestinal nematodes. Br. Poult. Sci..

[17] Stehr M., Sciascia Q., Metges C.C., Gauly M., Daş G. (2018). Co-expulsion of *Ascaridia galli* and *Heterakis gallinarum* by chickens. Int. J. Parasitol..

[18] Künstner A., Wolf J.B.W., Backström N., Whitney O., Balakrishnan C.N., Day L., Edwards S.V., Janes D.E., Schlinger B.A., Wilson R.K. (2010). Comparative genomics based on massive parallel transcriptome sequencing reveals patterns of substitution and selection across 10 bird species. Mol. Ecol..

[19] Chico V., Nombela I., Puente-Marín S., Ortega-Villaizan M.D.M. (2019). Nucleated red blood cells contribute to the host immune response against pathogens. Immune Response Activation and Immunomodulation.

[20] Yazwinski T.A., Chapman H.D., Davis R.B., Letonja T., Pote L., Maes L., Vercruysse J., Jacobs D.E. (2003). World Association for the Advancement of Veterinary Parasitology (WAAVP) guidelines for evaluating the effectiveness of anthelmintics in chickens and turkeys. Veter. Parasitol..

[21] Rahimian S., Gauly M., Daş G. (2016). Embryonation ability of *Ascaridia galli* eggs isolated from worm uteri or host faeces. Veter. Parasitol..

[22] Ferdushy T., Nejsum P., Roepstorff A., Thamsborg S.M., Kyvsgaard N.C. (2012). *Ascaridia galli* in chickens: Intestinal localization and comparison of methods to isolate the larvae within the first week of infection. Parasitol. Res..

[23] Kringel H., Roepstorff A., Murrell K.D. (2002). A method for the recovery of immature *Trichuris suis* from pig intestine. Acta Veter. Scand..

[24] Cringoli G., Maurelli M.P., Levecke B., Bosco A., Vercruysse J., Utzinger J., Rinaldi L. (2017). The Mini-FLOTAC technique for the diagnosis of helminth and protozoan infections in humans and animals. Nat. Protoc..

[25] Kaufmann J. (1996). Parasitic Infections of Domestic Animals: A Diagnostic Manual.

[26] Daş G., Hennies M., Sohnrey B., Rahimian S., Wongrak K., Stehr M., Gauly M. (2017). A comprehensive evaluation of an ELISA for the diagnosis of the two most common ascarids in chickens using plasma or egg yolks. Parasites Vectors.

[27] Bolger A.M., Lohse M., Usadel B. (2014). Trimmomatic: A flexible trimmer for Illumina sequence data. Bioinformatics.

[28] Trapnell C., Pachter L., Salzberg S.L. (2009). TopHat: Discovering splice junctions with RNA-Seq. Bioinformatics.

[29] Lawrence M., Huber W., Pagès H., Aboyoun P., Carlson M., Gentleman R., Morgan M.T., Carey V.J. (2013). Software for computing and annotating genomic ranges. PLoS Comput. Biol..

[30] Love M.I., Huber W., Anders S. (2014). Moderated estimation of fold change and dispersion for RNA-seq data with DESeq2. Genome Biol..

[31] Blighe K., Rana S., Lewis M. (2019). Enhanced Volcano: Publication-Ready Volcano Plots with Enhanced Colouring and Labeling. R Package Version 1.4.0. https://github.com/kevinblighe/EnhancedVolcano.

[32] Raudvere U., Kolberg L., Kuzmin I., Arak T., Adler P., Peterson H., Vilo J. (2019). g:Profiler: A web server for functional enrichment analysis and conversions of gene lists (2019 update). Nucleic Acids Res..

[33] Harrell F.E. (2020). with contributions from Charles Dupont and many others. Hmisc: Harrell Miscellaneous. R Package Version 4.3-1. https://CRAN.R-project.org/package=Hmisc.

[34] Wickham H. (2016). ggplot2: Elegant Graphics for Data Analysis.

[35] Alcorta D., Preston G., Munger W., Sullivan P., Yang J.J., Waga I., Jennette J.C., Falk R. (2002). Microarray studies of gene expression in circulating leukocytes in kidney diseases. Exp. Nephrol..

[36] Bull T.M., Coldren C.D., Moore M., Sotto-Santiago S.M., Pham D.V., Nana-Sinkam S.P., Voelkel N.F., Geraci M.W. (2004). Gene microarray analysis of peripheral blood cells in pulmonary arterial hypertension. Am. J. Respir. Crit. Care Med..

[37] Ma J. (2003). Gene profiling identifies secreted protein transcripts from peripheral blood cells in coronary artery disease. J. Mol. Cell. Cardiol..

[38] Ross R.W., Galsky M.D., Scher H.I., Magidson J., Wassmann K., Lee G.-S.M., Katz L., Subudhi S.K., Anand A., Fleisher M. (2012). A whole-blood RNA transcript-based prognostic model in men with castration-resistant prostate cancer: A prospective study. Lancet Oncol..

[39] Saenger Y., De Moll E., Fu Y. (2014). Transcriptional profiling of whole blood: A rich source of immune biomarkers in cancer. OncoImmunology.

[40] Meitern R., Andreson R., Hõrak P. (2014). Profile of whole blood gene expression following immune stimulation in a wild passerine. BMC Genom..

[41] Steffens N., Beuter-Gunia C., Kravets E., Reich A., Legewie L., Pfeffer K., Degrandi D. (2020). Essential Role of mGBP7 for Survival of *Toxoplasma gondii* Infection. mBio.

[42] Feng M., Zhang Q., Wu W., Chen L., Gu S., Ye Y., Zhong Y., Huang Q., Liu S. (2021). Inducible guanylate-binding protein 7 facilitates influenza a virus replication by suppressing innate immunity via NF-kappab and JAK-STAT signaling pathways. J. Virol..

[43] Yazdanpanah B., Wiegmann K., Tchikov V., Krut O., Pongratz C., Schramm M., Kleinridders A., Wunderlich T., Kashkar H., Utermöhlen O. (2009). Riboflavin kinase couples TNF receptor 1 to NADPH oxidase. Nat. Cell Biol..

[44] Nam S., Lim J.-S. (2016). Essential role of interferon regulatory factor 4 (IRF4) in immune cell development. Arch. Pharmacal Res..

[45] Ruhnke I., Andronicos N.M., Swick R.A., Hine B., Sharma N., Kheravii S.K., Wu S.-B., Hunt P. (2017). Immune responses following experimental infection with *Ascaridia galli* and necrotic enteritis in broiler chickens. Avian Pathol..

[46] Daş G., Hennies M., Tuchscherer A., Gauly M., Matthias G. (2018). Time- and dose-dependent development of humoral immune responses to *Ascaridia galli* in experimentally and naturally infected chickens. Veter. Parasitol..

[47] Allen J.E., Maizels R.M. (2011). Diversity and dialogue in immunity to helminths. Nat. Rev. Immunol..

[48] Råberg L. (2014). How to live with the enemy: Understanding tolerance to parasites. PLoS Biol..

[49] Kim B.-H., Shenoy A.R., Kumar P., Das R., Tiwari S., MacMicking J.D. (2011). A family of IFN- -Inducible 65-kD GTpases protects against bacterial infection. Science.

